# How risky is it to visit a supermarket during the pandemic?

**DOI:** 10.1371/journal.pone.0253835

**Published:** 2021-07-01

**Authors:** Alexey A. Tsukanov, Alexandra M. Senjkevich, Maxim V. Fedorov, Nikolai V. Brilliantov

**Affiliations:** 1 Center for Computational and Data-Intensive Science and Engineering, Skolkovo Institute of Science and Technology, Moscow, Russia; 2 Department of Mathematics, University of Leicester, Leicester, United Kingdom; Univerza v Mariboru, SLOVENIA

## Abstract

We performed large-scale numerical simulations using a composite model to investigate the infection spread in a supermarket during a pandemic. The model is composed of the social force, purchasing strategy and infection transmission models. Specifically, we quantified the infection risk for customers while in a supermarket that depended on the number of customers, the purchase strategies and the physical layout of the supermarket. The ratio of new infections compared to sales efficiency (earned profit for customer purchases) was computed as a factor of customer density and social distance. Our results indicate that the social distance between customers is the primary factor influencing infection rate. Supermarket layout and purchasing strategy do not impact social distance and hence the spread of infection. Moreover, we found only a weak dependence of sales efficiency and customer density. We believe that our study will help to establish scientifically-based safety rules that will reduce the social price of supermarket business.

## Introduction

Coronavirus 2019 disease (COVID-19), which is caused by the severe acute respiratory syndrome coronavirus 2 (SARS-CoV-2) was first reported in humans in late 2019 and the World Health Organization declared it a pandemic in March 2020. By May, 2021 it had infected over 170 million globally, including a reported about 34 million in USA, 28 million in India and 17 million in Brazil [1].

The coronavirus spreads by contact and through airborne respiratory droplets and a complete lockdown, which enforces isolation and social distancing, is the most effective means to slow the spread. Unfortunately, the economic impact of such lockdowns is devastating and lockdowns are untenable for extended periods of time. Additionally, a complete lockdown is impractical since people need to purchase food, medicines and basic supplies. Many types of stores are considered essential service providers during a pandemic including supermarkets, smaller markets, and pharmacies. These stores, while providing necessary services, should also be allowed to earn a profit that benefits the economy. However, while these stores should remain open, they must conduct their business in the most efficient manner to reduce the spread of the infection. Thus, how these essential businesses function is a trade off between earned profit and potential for increased infection. We propose a model that examines scientifically-based safety rules for the supermarket business and assesses the social price of such an economic activity.

Fortunately, the general mechanisms of the disease transmission are presently well documented allowing rules governing visits to places (like supermarkets) that may become crowded [2, 3]. The most existing models regarding human behavior and infection spread analyze the global aspect of an epidemic, that is, the infection spread “in average” or on a “geographic” space-scale, see for example [4–13]. Neither the statistics of inter-personal contacts, nor a particular geometry of a place where people may be infected, have been studied. Naturally, safety rules should be deduced from a microscopic model of inter-personal contacts [14, 15]; still such microscopic models are presently lacking, see e.g. the review Ref. [16] for the current state of mathematical epidemiology.

Here we elaborate a microscopic model of the infection transmission. It explicitly accounts for individual inter-personal contacts and a geometry of a space—a supermarket. We explore how safe it could be to visit a supermarket. That is, we pose a question, how many people could simultaneously visit a supermarket, keeping an acceptable infection rate. This may be done using an adequate mathematical model describing the behavior of customers and the infection transmission between them. Quantitatively speaking, we need to assess the probability for a visitor to get infected, depending on a number of customers in a supermarket and on a percentage of infected among them. This quantity may also depend on the purchasers behavior, and on the geometry of the place.

The purchasers behavior comprises two different components: (i) mutual interactions between purchasers and (ii) their strategy in the supermarket. The mutual interactions may be rather adequately modeled by the so-called “social force model”, proposed by Helbing et al. [17]. It realistically reproduces various phenomena in people flows [18], including lane formation in bidirectional pathway, oscillations at bottlenecks, blocked states in emergency situations and self-organization of a crowd. Moreover, the behavior of a crowd in a complex geometry, and even three-dimensional interactions at staircases [19, 20] may be also reproduced. The social-force-based simulations require a relatively small number of parameters and prove to be applicable for a wide range of social situations [18] (see [21] for a recent review).

On the contrary, the strategy of a customer is associated with a particular place. It will be different in a supermarket or in a small store, in a restaurant or in an airport. Therefore, the development of a model, that realistically mimics a strategy of a customer in different places, is an important component of the such models, which dictates the safety rules. Presently a number of models of a customer strategy is available, starting from the old intervening-opportunities model, first proposed by Stouffer in 1940 [22]. These models, as well as their modifications have been used in numerous applications, including the analysis of intra-city mobility, interstate migration, etc., see e.g. [23–26]. The other class of model are the so-called gravity [27–29] or radiation [30–32] models. A comprehensive review of the application of the different models to the customer mobility in supermarkets may be found in Ref. [33]. All these models, however, being efficient in solving many problems, lack some features, important for modeling the infection transmission. Namely, the features that determine how long a customer resides in a supermarket and what is the distribution of inter-customer distances. In the present study we develop a model that possess the needed features. In short, it mimics the purchase goals and psychological aspects of a customer which is detailed in the next sections.

Finally, a *microscopic* person-to-person infection transmission model is to be formulated. In a recent study [15] a simple model has been proposed that relates the probability of getting infected with the duration of time spent by a healthy person in a zone around an infected one. Here we introduce a similar, but a somewhat more realistic model. It takes into account that the probability of getting infected depends both, on the time interval, as well as on the distance between people; we use a continuous function to describe this dependence.

Since direct experimental data for the infection spread on microscopic (i.e. individual) level are presently lacking, we are not able to calibrate our model. Nevertheless, the model allows to perform a general analysis and answer the following important questions: (i) what is the probability for a customer to get infected in a supermarket and how it depends on a number of customers in the store and on the percentage of infected visitors; (ii) how sensitive is this probability to the social distance between the customers and what is the optimal social distance, and finally, (iii) what is the impact of a supermarket geometry on the probability to get infected. To answer the above questions we have performed large-scale simulations based on the above composite model. The results will help to answer, what could be done to reduce the infection rate and hence the social price of the supermarket business.

The rest of the article is organized as follows. In the next section we consider results, including the elaborated model and outcomes of numerical simulations. In third section we discuss and summarize the obtained results. Technical details are presented in the Materials and Methods and S1 Appendix.

## Composite model

### Social forces

To describe the customer motion we use the concept of social forces applied to model pedestrian fluxes [17, 34]. The main idea of this concept is that pedestrians change their velocity depending on the location and velocity of the surrounding objects, which may be other pedestrians, walls, columns, etc. The change of the velocity, that is the acceleration, may be put in a form of the Newton’s second law with fictitious “forces”. This yields the equations of motion for *i*th pedestrian:

$$\begin{array}{ccc} \frac{dr_{i}}{dt} & = & v_{i} \end{array}$$ (1)

$$\begin{array}{ccc} \frac{dv_{i}}{dt} & = & F_{i}^{\text{des}}+F_{i}^{\text{soc}}+F_{i}^{\text{fluc}}+F_{i}^{\text{chir}}+F_{i}^{\text{obs}}. \end{array}$$ (2)

Here **r**_*i*_ and **v**_*i*_ are, respectively, the radius vector and velocity of the pedestrian (customer). The total force is comprised of different parts, which mimic the most prominent features of a pedestrian behavior, with the parameters calibrated on observations, see e.g. [17, 34]. The first term of the right hand side of Eq (2) **F**^des^ reflects the tendency of a pedestrian (we skip the index *i* for brevity) to move with a certain desired velocity:

$$\begin{array}{c} F^{\text{des}}=\tau^{-1}(v^{\text{des}}-v), \end{array}$$ (3)

where *τ* is the relaxation time, **v**^des^ is a vector of the desired velocity, which absolute value is fixed and the direction may change subjected to the strategy; for physiological reasons the desired velocity is limited from the above, *v*^des^ ≤ *v*^max^. Here we use the calibrated values of *τ* = 0.5 s and *v*^des^ = 1.34 m/s with *v*^max^ = 1.3 · *v*^des^ from Ref. [17, 35].

The second term describes interactions between pedestrians at locations (*x*_*i*_, *y*_*i*_) and (*x*_*j*_, *y*_*j*_) [17, 34]:

$$\begin{array}{c} F_{x}^{\text{soc}}=Ae^{-\epsilon /B}\frac{1}{2}\frac{x_{i}-x_{j}}{d}(1-c_{x}\frac{x_{i}-x_{j}}{d}) \end{array}$$ (4)

$$\begin{array}{c} F_{y}^{\text{soc}}=Ae^{-\epsilon /B}\frac{1}{2}\frac{y_{i}-y_{j}}{d}(1-c_{y}\frac{y_{i}-y_{j}}{d}) \end{array}$$ (5)

where $d=\sqrt{{\left(x_{i}-x_{j}\right)}^{2}+{\left(y_{i}-y_{j}\right)}^{2}}$, *ϵ* = *d* − 2*R* and *R* gives the “social radius” of a pedestrian. $c_{x}=v_{xi}/\sqrt{v_{xi}^{2}+v_{yi}^{2}}$, $c_{y}=v_{yi}/\sqrt{v_{xi}^{2}+v_{yi}^{2}}$ are the dimensionless components of the velocity vectors of *i*th pedestrian. Following the Ref. [17] we use *B* = 0.3 m for the relaxation distance and *A* = 2.1 m/s^2^ for the force amplitude. Here we vary the social radius *R*, which mimics the tendency of people to keep larger inter-personal distance during the pandemic; the value of *R* = 0.2 m was used in Ref. [17].

The third term **F**^fluc^ quantifies unavoidable randomness of the pedestrian motion, modeled by a fluctuation force—a Gaussian white noise with zero mean. That is, $\langle F_{i}^{\text{fluc}}\rangle =0$ and $\langle F_{i}^{\text{fluc}}\left(t\right)F_{j}^{\text{fluc}}\left(t^{\prime }\right)\rangle =\bar{F^{2}}\delta_{ij}\delta \left(t-t^{\prime }\right)$; here we use $\bar{F^{2}}=0.01$.

The fourth term of the social force refers to the chirality of inter-personal interactions which allows to explain lane formation [36]. We assume that if pedestrians are approaching each other, each of them tends to turn *right*, avoiding the collision:

$$\begin{array}{c} F^{\text{chir}}=\chi \Theta (v_{ij}\cdot r_{ij})\Theta (-v_{i}\cdot v_{j})\Theta (D-d)N, \end{array}$$ (6)

where Θ(x) is the Heaviside step function, **N** is a unit vector, perpendicular to the inter-pedestrian distance **r**_*ij*_ = **r**_*i*_ − **r**_*j*_; it shows the direction of motion corresponding to the turn to the right. The first step function in the r.h.s. of the above equation guarantees that two pedestrians approach each other, the second function—that they move in opposite directions. Finally, the third step function indicates that the pedestrians are within the range distances where the chirality force acts. The unit vector **N** may be written as **N** = (**r**_*ij*_ × **v**_*ij*_) × **r**_*ij*_/|(**r**_*ij*_ × **v**_*ij*_) × **r**_*ij*_|. In our simulations we assume that the chirality factor is χ = 0.14 m/s^2^ and the according distance is *D* = 4 m [36].

The last term in the r.h.s of Eq (2) describes interactions of a customer with walls and obstacles. In Ref. [17] the following form of the force from a wall or an obstacle has been proposed:

$$\begin{array}{c} F_{i}^{obs}=-\nabla_{r_{i\;w}}U_{i\;w}\left(\right|r_{i\;w}\left|\right), \end{array}$$

where **r**_*iw*_ = **r**_*i*_ − **r**_*w*_ and **r**_*w*_ is the position of that part of the wall, which is the nearest to *i*th pedestrian. *U*_*iw*_(*r*) is the according potential of the wall, modeled as an exponential [17].

**Fig 1:**
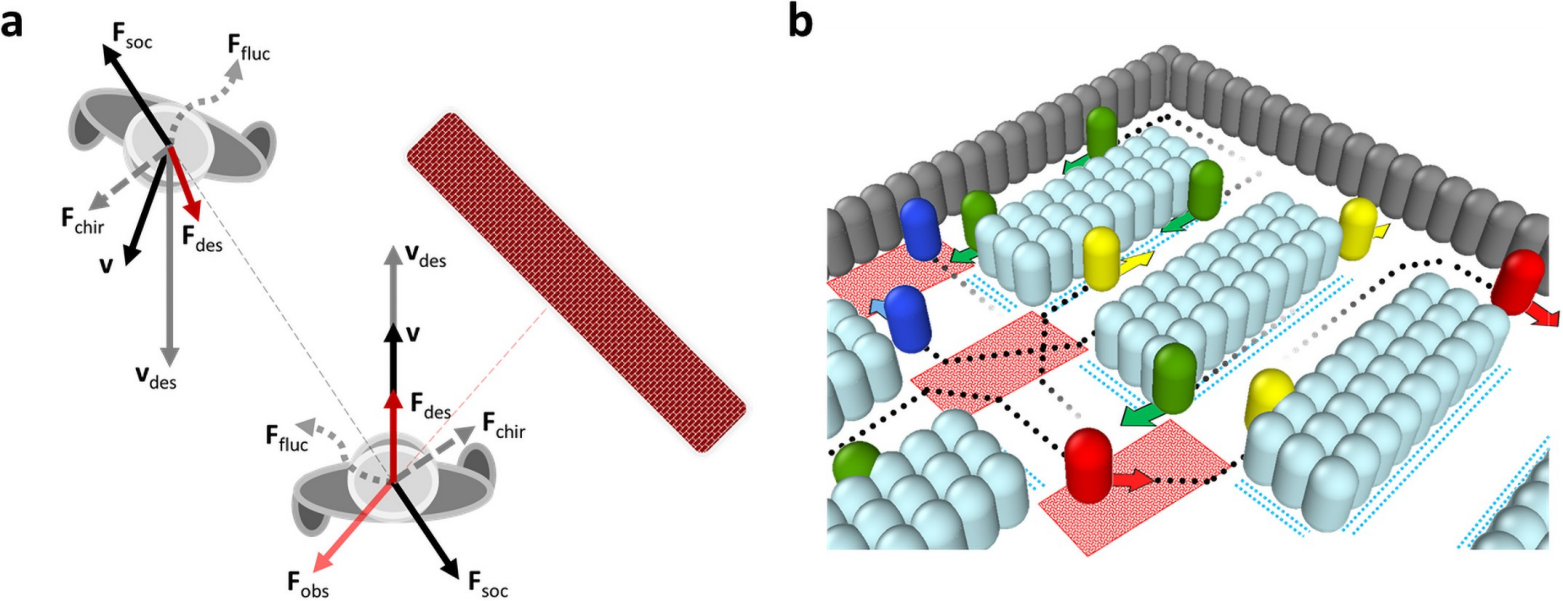
Illustration of the components of the composite mode: (a) Social force model—a schematic diagram of the terms of Eq (2). (b) Sketch of the customers strategy—the behavior in the crossroad zones and near shelves. The examples of the desired (not actual) trajectories are shown. The notations are: red area—crossroad zone, blue dashes—slow motion zone (near the shelves). Gray cylinders indicate walls, light-blue—shelves with goods. The colour of visitor (cylinders) encodes the desired direction of motion: North (blue), South (red), East (yellow), and West (green), see also S1 Appendix.

Real walls or obstacles, however, correspond to a hard-core potential, hence a realistic modeling requires a rather steep potential *U*_*iw*_(*r*). This is not computationally convenient as it requires too small time step when a pedestrian is close to a wall. Therefore, instead of modeling the interaction of a pedestrian with a wall with a smooth potential, *U*_*iw*_(*r*), we consider “collisions” of pedestrians with the walls or obstacles. This significantly simplifies computations and corresponds to the so-called “event-driven” modeling of granular matter, instead of force-driven modeling, see e.g. [37]. We implemented the collision rule as follows. Let **r**_*i*_(*t*) be the position of *i*th customer at time *t*, and the calculated position at time *t* + *dt* be **r**_*i*_(*t* + *dt*) (*dt* is the computational time step). Let **r**_*i*_(*t* + *dt*) belong to an inaccessible area. Then the position persists, **r**_*i*_(*t* + *dt*) = **r**_*i*_(*t*), while the velocity is updated according to the collision rule:

$$\begin{array}{c} v_{i}\left(t+dt\right)=\left(v_{i}\left(t\right)\cdot t\right)t-\xi \left(v_{i}\left(t\right)\cdot n\right)n, \end{array}$$ (7)

where 0 < *ξ* < 1 is the velocity recovery factor, **N** is the unit vector of external normal to the wall and **t** is the tangential one. In our simulations we use *ξ* = 0.1. Eq (7) describes a damped reflection of the normal component of the velocity and persistence of the tangential component; in the context of granular matter it corresponds to inelastic collision of a smooth particle [37]. A schematic diagram of the terms in the equations of motion Eq (2) is given in Fig 1.

Note that the social forces model assumes the homogeneity of customers in terms of their social distancing practices. Certainly this is an oversimplification, since humans differ in their actions. Still, as long as average properties of a large group of people are addressed, this is not important: variations in the individual behavior, associated e.g. with the inter-personal distance, are averaged out. Hence for the aims of our study, the social force model, calibrated on numerous real-life phenomena, is adequate.

### Customer strategy

For realistic simulations of customers behavior in a supermarket it is necessary to formulate a customer strategy. The strategy comprises a set of locomotion rules which govern the agents to change or to keep the direction of the desired velocity **v**_des_. The rules depend on a couple of factors, which we discuss in what follows.

#### The dependence of v_des_ on the location

**Fig 2:**
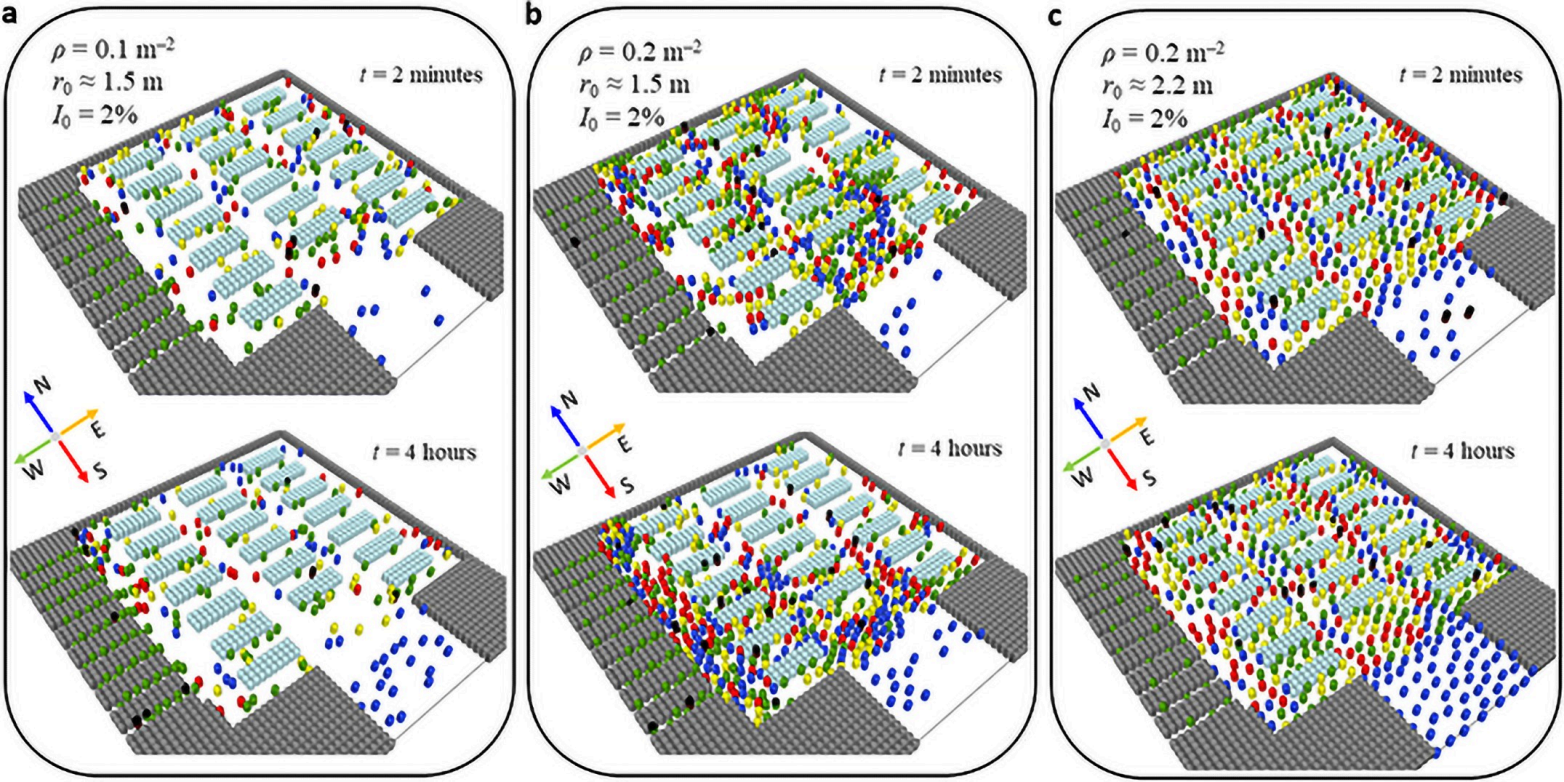
Snapshots of the studied system for different customer density *ρ*—The average number of visitors per unit area and desired social distance *r*_0_ for 2 minutes and 4 hours after the system initialization. The percentage of initially infected people *I*_0_ is 2%. Color code for pedestrian: blue—**v**_des_ is directed to North (N), red—South (S), yellow—East (E), green—West (W), deep purple with black cap—initially infectious visitor, and with black cap—infected.

The first set of rules prescribes a customer direction and a magnitude of **v**_des_ for different zones:

1. **v**_des_ in the entrance zone is directed along the entrance lanes used to enter the supermarket (North direction for the studied setup, see Fig 2). In addition, the magnitude of *v*_des_ in the entrance zone is scaled by a factor *k*_*E*_ = 0.05 (that is, we use *k*_*E*_ *v*_des_ in this zone). Such a small scaling factor for the velocity *v*_des_ in the entrance zone is used to simulate individuals queueing to enter; this prevents also crowding at the entrance when the visitor flux becomes too high.
2. **v**_des_ in the zone of queue at the cashier desks is directed along the line of exit from the supermarket (West direction for the studied setup, see Fig 2). The factor for the magnitude of *v*_des_ in a queue is *k*_*Q*_ = 0.03.
3. In order to account for the slowdown in the customers movement near the shelves with products, a scaling factor *k*_*S*_ = 0.3 was used for desired velocity *v*_des_.
4. **v**_des_ in all other places is uniformly distributed over the four cardinal directions (North, East, South and West) and is not scaled.

The change of the **v**_des_ direction occurs, if a customer approaches a wall or an obstacle, if he/she enters the “crossroads” zones (which are mainly the crosses, see Fig 2), or if he/she is not able to move with the desired velocity. The change of the **v**_des_ depends also on the purchasing stage detailed below.

#### Purchasing behavior and purchasing stage

The purchasing stage may be either complete (C) or incomplete (Ic). In the former case all planned purchases have been made, in the later one—some purchases are still to be done. The purchasing behavior obeys a set of rules.

Each customer which enters the supermarket receives an individual *shopping list*—a random number of purchases *π*_*i*_ to buy. This Gaussian distributed number, with the mean 〈*π*〉 and standard deviation *σ*_*π*_, is defined for each visitor. The counter of purchases $c_{i}^{\pi }$ is also defined for each *i* and initially set to zero. Furthermore, if point of predicted position **r**_*p*_(*t* + *t*_p_) = **r**(*t*) + **v**^des^*t_p_* is a shelving with goods, the purchases counter increases $c_{i}^{\pi }:=c_{i}^{\pi }+1$. The initial purchasing stage of a customer is incomplete (Ic). When, however, $c_{i}^{\pi }⩾\pi_{i}$, the purchasing stage is converted to become complete (C). Customers in different stages C or Ic obey different rules for changing **v**_des_, as it discussed below. In our model, the mean number of purchases is 〈*π*〉 = 40 units, and the standard deviation is *σ*_*π*_ = 20 units.

#### Rules for the change of v_des_

The change of the **v**_des_ direction takes place when

1. A customer approaches a wall or obstacle.
  Each time step a customer *predicts* his/her future position after time *t*_*p*_ **r**_*p*_(*t* + *t*_p_) = **r**(*t*) + **v**^des^*t_p_*. If this point belongs to an area beyond a wall or inside an obstacle, the customer revises the direction of his/her movement. With the probability of 0.4 the customer decides to turn left, with the same probability to turn right, and with the probability of 0.2—to make an U-turn. The forward prediction time *t*_*p*_ = 1.5 s was used in our simulations.
2. A customer, in a purchase stage Ic, enters “crossroads” zones (Rules A)
  If a pedestrian that continues the purchasing, enters a crossroad zone, he/she decides with some probability *P*_cd_ to change the direction. Namely, he/she turns right, or left with the equal probabilities $\frac{1}{2}P_{\text{cd}}$, or continues to go straight ahead with the probability (1 − *P*_cd_). If the decision to change the direction is made, then this is performed in two steps. Firstly, when the customer enters the crossroad zone—the desired velocity **v**_des_ changes by 45° in the direction chosen. Secondly, when he/she exits the crossroad zone—the direction of **v**_des_ additionally changes by 45°; this results in a complete turn by 90°. This is illustrated in Fig 1, see also S1 Fig in S1 Appendix. The modulus of the vector **v**_des_ remains unchanged. We used *P*_cd_ = 2/3 in our model. Examples of desired, as well as actual customers trajectories are shown in S1 Fig in S1 Appendix.
3. A customer, in a purchase stage C, enters “crossroads” zones (Rules B)
  If a customer that has completed the purchasing, enters a crossroad zone, he/she obeys the following rules. If a customer enters a crossroad zone from the southern (S) or northern (N) direction (recall that the cashier desks are located at the West border, see Fig 2), he/she makes a decision with a probability of *P*_*cd*_ = 2/3 to turn towards the cashier desks, otherwise he/she continues to move straight. If a customer enters the crossroad, moving away from the cashier desks, he/she turns by 90° to the left or to the right (i.e. to S or to N), with the probability of *P*_*cd*_; otherwise with the probability (1 − *P*_*cd*_) he/she turns towards the cashier desks (i.e. toward W) and exits. If a customer enters the crossroad zone, moving towards the cashier desks and exits (i.e. toward W), he/she turns by 90° left or right (i.e. to S or N) with the probability of *P*_cd_, or persists moving with the probability (1 − *P*_cd_) in the same direction.
4. A customer lacks patience to stand and wait, or to move too slowly.
  For a more realistic description of people’s behavior in a case of congestion, which hinders their motion in the desired direction, we have introduced a counter of customer patience. It is implemented as follows:
  - A float-value counter of patience Π_*i*_ is defined for each *i*th pedestrian, the unit of patience Π_*i*_ is a time unit (second), an initial value is zero.
  - At each timestep *t* the scalar product of *i*th customer actual speed **v**_*i*_ and the desired one $v_{i}^{\text{des}}$ is calculated. If at the current step the inequality
    $$\begin{array}{c} \left(v_{i}\cdot v_{i}^{\text{des}}\right)<k_{d}{\left(v_{i}^{\text{des}}\right)}^{2}, \end{array}$$ (8)
    where the coefficient *k*_*d*_ < 1 is a downtime factor, is satisfied, then the patience counter Π_*i*_ of *i*th is increased by timestep *dt* (Π_*i*_ ≔ Π_*i*_ + *dt*), else the counter is reset to zero Π_*i*_ ≔ 0.
  - If the patience of the customer is *overflowed*, i.e. reaches a threshold value Π^max^, the customer revises the direction of his/her movement $v_{i}^{\text{des}}$: Equally with, a probability of 1/4, the pedestrian either continues trying to move in previously desired direction, or chooses a new one from the other of the three remaining cardinal directions. After that the patience counter of *i*th pedestrian is reset to zero Π_*i*_ ≔ 0.
  The introduction of such a counter into the model allows a pedestrian which stuck in a congestion to “change their mind” and go back or go around it. We believe that this is an important feature of a customer strategy, which allows an adequate modeling, especially, when the number of customers in a supermarket is not small. The downtime factor *k*_*d*_ = 0.2 and Π^max^ = 7 s were used in our simulations.
5. A customer gets to the end of the queue to the cashier or arrives at the exit
  In this case the customer is removed from the supermarket and another customer randomly added to the entrance, to keep the average density of customers constant.

The elements of the customer strategy are illustrated in Fig 1.

### Model of the infection transmission

We assume that initially *I*_0_% of customers in the supermarket are infected and can infect other visitors. Let *j*th pedestrian be infective. We propose the following model of the infection transmission for two pedestrians with the coordinates **r**_*i*_, **r**_*j*_ and velocities **v**_*i*_, **v**_*j*_:

$$\begin{array}{c} P_{\text{inf}}=[A_{\text{iso}}^{\text{inf}}+A_{\text{aniso}}^{\text{inf}}\Theta (-\left(v_{j}-v_{i}\right)\cdot r_{ij})]k_{j}^{\text{mask}}e^{-r_{ij}/\kappa }, \end{array}$$ (9)

where ***P***_inf_ is the probability to transmit the infection per unit time (i.e. ***P***_inf_ *dt* gives the probability to get infected during the time interval *dt*). It comprises two components—the isotropic and anisotropic one, and $A_{\text{iso}}^{\text{inf}}$, $A_{\text{aniso}}^{\text{inf}}$ are the respective non-negative coefficients. The former term, with $A_{\text{iso}}^{\text{inf}}$, describes the isotropic spread of infection, independently of the relative velocities of two individuals. The latter one, with $A_{\text{aniso}}^{\text{inf}}$, describes the enhanced infection transmission when the pedestrians move towards each other; in this case the unit Heaviside step function Θ(⋅) is non-zero. **r**_*ij*_ = **r**_*i*_ − **r**_*j*_ denotes the inter-pedestrian vector and **v**_*ij*_ = **v**_*i*_ − **v**_*j*_—their relative velocity. The coefficient $k_{j}^{\text{mask}}$ quantifies the reduction of the infection transmission by a medical mask (mask factor). It equals 1, if an infectious pedestrian *j* does not wear a mask and and $k_{j}^{\text{mask}}=c^{\text{mask}}<1$, if the mask is used. Finally, *κ* is the characteristic infection-transmission length.

The above model is physically motivated and based on the airborne transmission mechanism, which has a strong support for the case of COVID-19 [3, 38, 39]. Indeed, the probability to get infected depends on the amount of the infectious substance received by a healthy person in a contact with an infectious one. The infectious person exhales permanently air with aerosol droplets containing the virus. Hence, the amount of transmitted infectious substance (droplets with virus) is proportional to the duration of the inter-person contact [15] and concentration of the infected aerosol inhaled by the healthy person. Obviously, this concentration decays with the increasing distance *r*_*ij*_ from the infectious individual. To obtain a functional form of this dependence is very challenging, as multiple random processes are involved. Therefore, we adopt here a computationally convenient exponential model. That is, we assume that the probability to get infected per unit time (associated with the concentration of inhaled aerosol) decreases with the increasing inter-personal distance *r*_*ij*_ exponentially. The characteristic length *κ* quantifies this decay. Furthermore, according to the available statistics, the wearing a medical mask reduces the probability of contracting various respiratory infections, including COVID-19, by a factor of about 1.8 [40]. Based on this we set the mask factor *c*^mask^ = 0.5.

As it commonly accepted now, respiratory infections can be transmitted through droplet particles which diameter is order of micrometers (*μm*) [41]. The droplet particles with a size less than 5 *μm* can remain in the air for a long period of time, transmitting the infection over distances more than 1 m, while larger particles with a diameter of the order ∼ 10 *μm* (so called, “respiratory droplets”) can transmit the infection if the inter-personal distance is about 1 m or less [42]. Data on COVID-19 transmission indicates that the virus is primarily transmitted between people through respiratory droplets and contact routes [38, 39, 43, 44]. For this reason, the characteristic distance of person-to-person infection transmission, we used in our model, has an order of 1 m. For the sake of simplicity and due to the lack of data to parameterize the infection transmission we also used $A_{\text{iso}}^{\text{inf}}=0.01$, $A_{\text{aniso}}^{\text{inf}}=0$. Note that neglecting the anisotropic term we do not expect that it has a significant impact. Moreover, we expect that the contribution of this term may be accounted by re-normalizing the isotropic factor $A_{\text{iso}}^{\text{inf}}$.

With the chosen characteristic length *κ* = 2/ln 100 ≈ 0.4343 m the probability to transmit an infection decreases by a factor of 100 at 2 m distance. Additionally, a cutoff distance for infection transmission $r_{\text{cut}}^{\text{inf}}=4$ m was utilized, since respiratory droplets can hardly overcome such a distance.

Only initially infected people can infect other visitors of the supermarket, and newly infected people are not infectious. In our simulations approximately half of infectious visitors wear medical mask.

Although the above parameters of the infection transmission model are not calibrated to COVID-19 we expect that with the use of these parameters the most prominent features of the infection transmission in crowded places may be revealed. Indeed, the chosen parameters comply with the observed characteristic lengths for spreading of aerosol droplets carrying the infection.

### Simulation detail

We considered the system with a constant number of people *N* in the supermarket. Hence the average number density of customers ρ = *N*/*S*_shop_ (*S*_shop_ is the total area of the supermarket excluding shelf and wall areas) is constant, *ρ* = const. At the first stage of the system initialization, the number of visitors *N* = *ρ* *S*_shop_ was set, corresponding to the chosen values of density *ρ*.

We designed three supermarket models having different geometry; a typical supermarket geometry used in our simulations is depicted in Fig 2. The other schemes and their detailed description are provided in the S3 and S4 Figs in S1 Appendix. At the initialization stage the customers are uniformly distributed over the free area of the supermarket.

For the numerical integration of the equations of motion (1) the simple Euler algorithm has been used. This algorithm is fast and not computationally expensive, since the pedestrian velocity is limited by 1.3 of *v*_des_ and the forces are relatively soft. Program realization of the algorithm including post-processing was made using C/C++ programming language (the developed code with all necessary data files are available via link https://github.com/AATsukanov/Infection-Transmission-Model-2021), a 3D-visualization of the results was performed with OVITO package [45].

Each point in the plot(s) corresponds to a single run of 144 000 time steps with *dt* = 0.1 s, which corresponds to the modelling of the supermarket for about 4 hours. The reported values are typically the time-averaged quantities over the last half of a simulation run.

Simulation detail and the values of the parameters of the model can be found in Table 1 as well as in S1 Appendix.

**Table 1:** Parameters of the composite model.

Parameter	Value	Description
*τ*	0.5 s	velocity relaxation time, Eq (3)
*v*^des^	1.34 m/s^2^	module of the desired velocity, Eq (3)
*v*^max^	1.3 · *v*^des^	maximal actual velocity
*k*_*E*_	0.05	factor for *v*^des^ in the entrance zones (“E”)
*k*_*Q*_	0.03	factor for *v*^des^ in a queue (“:”)
*k*_*S*_	0.3	factor for *v*^des^ in slow motion zones (“.”)
*ξ*	0.1	velocity recovery factor, Eq (7)
*A*	2.1 m/s^2^	inter-person repulsion force amplitude, Eqs (4) and (5)
*B*	0.3 m	inter-person relaxation distance, Eqs (4) and (5)
*χ*	0.14 m/s^2^	magnitude of the chirality force, Eq (6)
*D*	4 m	chirality force cutoff distance, Eq (6)
〈*π*〉	40 units	mean number of purchases in the purchase list
*σ*_*π*_	20 units	standard deviation for number of purchases
*t*_*p*_	1.5 s	forward prediction time (see Rules for the change of **v**_des_)
*P*	0.4	probability to turn left/right, approaching wall or obstacle
*P*_cd_	2/3	probability to change the direction in a crossroad zone
*k*_*d*_	0.2	downtime factor for a customer patience, Eq (8)
Π^max^	7 s	the threshold level of a customer patience
${\text{A}}_{\text{iso}}^{\text{inf}}$	0.01	coefficient for isotropic term, Eq (9)
${\text{A}}_{\text{aniso}}^{\text{inf}}$	0	coefficient for anisotropic term, Eq (9)
*κ*	0.4343 m	characteristic infection-transmission length, Eq (9)
*c*^mask^	0.5	mask factor, Eq (9)
		probability to transmit infection, Eq (9)
$r_{\text{cut}}^{\text{inf}}$	4 m	a cutoff distance for infection transmission
*dt*	0.1 s	integration time step
*T*	4 h	simulation run duration (144000 timesteps)

## Results and discussion

### The distribution of inter-customer distances

**Fig 3:**
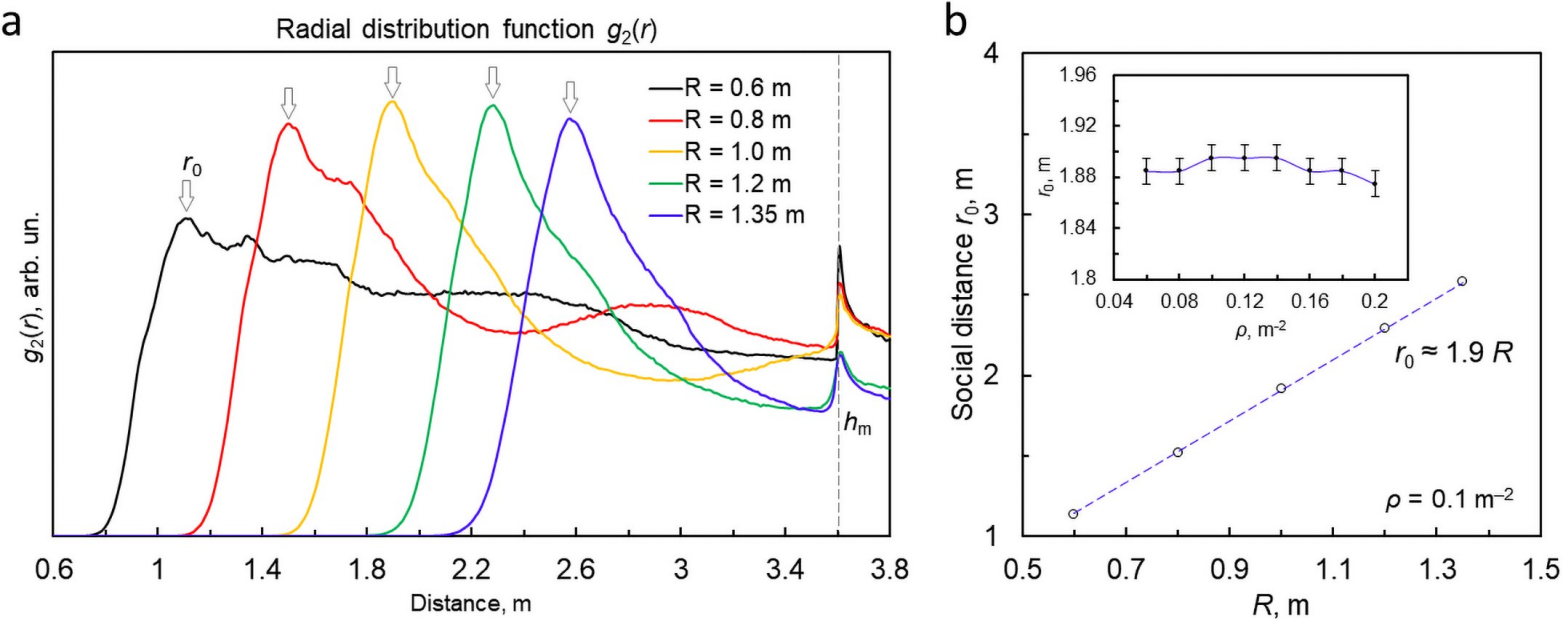
The distribution of inter-customer distances specified by the radial distribution function *g*_2_(*r*) for different social radii *R*. (a) The *g*_2_(*r*) for the typical density *ρ* = 0.1 *m*^−2^; *h*_*m*_ indicates the passage width. (b) The dependence of the peak location *r*_0_ (indicated in panel (a)) on *R*. The least square fit gives *r*_0_ ≃ 1.9 *R* for *ρ* = 0.1 *m*^−2^. With increasing density *ρ* the peak location slightly shifts towards smaller distances, which may be neglected for the addressed range of parameters (see the inset).

The average distance between customers is one of the key factors to control the infection transmission. This distance is mainly determined by the *social radius* *R* and pedestrian density *ρ*. Recall that the social radius *R* in Eqs (4) and (5) determines the strength of the inter-person repulsion, that is, an intention of a person to keep apart from another one. Due to many factors the interpersonal distance is a randomly varying quantity. Hence it is worth to analyze its statistical distribution. It is quantified by *g*_2_(*r*), so that the average number of customers within the distance interval (*r*, *r* + *dr*) from a randomly chosen customer reads *ρg*_2_(*r*)*dr*. This function is defined in the same way as a pair distribution function in the condensed matter physics, see e.g. [46]. For the two-dimensional case, addressed here, this function was computed as follows. For each customer *i* (*i* = 1, …, *N*) a number *n* of other customers *j* (*i* ≠ *j*) inside a ring of radius *r* and thickness *dr*, have been computed and divided by the area of the ring. The averaging over all customers *i* was then performed. The peak of *g*_2_(*r*) at *r* = *r*_0_ characterizes the most probable distance between two nearest customers. In what follows we call *r*_0_ the *“social distance”*. Note that while *R* is a model parameter, *r*_0_ may be measured in experiments, therefore the value *r*_0_ will be used in the discussion below, instead of *R*. Fig 3 illustrates the dependence of *g*_2_(*r*) and *r*_0_ on the density and social radius.

As it may be seen from the Fig 3, at the intermediate customer density *ρ* = 0.1 *m*^−2^ the most probable distance between customers increase with the social radius. The mean-least square fit yields the linear dependence,

$$\begin{array}{c} r_{0}(R)≃1.9\;R\;\;\text{for}\;\rho =0.1\;m^{-2}. \end{array}$$

At the same time a rather weak dependence of *r*_0_ on the density has been observed: While the density varies by a factor of more than 3 (from *ρ* = 0.06 *m*^−2^ to *ρ* = 0.20 *m*^−2^), the values of *r*_0_ varies only by about 5% (see the inset in Fig 3b).

Note, that from everyday observations one expects that the average distance between customers would be inversely proportional to their density. This expectation refers, however, to the case of large densities, when it is not possible to maintain a comfortable social distance inside a crowd. In pandemic times, such high densities are not achieved due to imposed restrictions on the number of customers in a shopping area. For this reason, we limited density *ρ* from above by 0.2 m^−2^, which generally speaking, is marginal with respect to safety. In this range of densities, the desired social distance $r_{0}^{max}$ is practically independent on *ρ*, which is clearly seen in the inset of Fig 3b.

### Time spent in a supermarket

The time spent by a customer in a supermarket is an important factor for the infection spread. Hence it is worth to know its dependence on various parameters. Besides, this quantity may indicate the adequacy of the model. Indeed, it may be compared with the everyday observation value, expected to be of the order of tens of minutes. The time spent in a supermarket comprises a time spent in shopping room and a time spent at the cash desk, including a queue.

**Fig 4:**
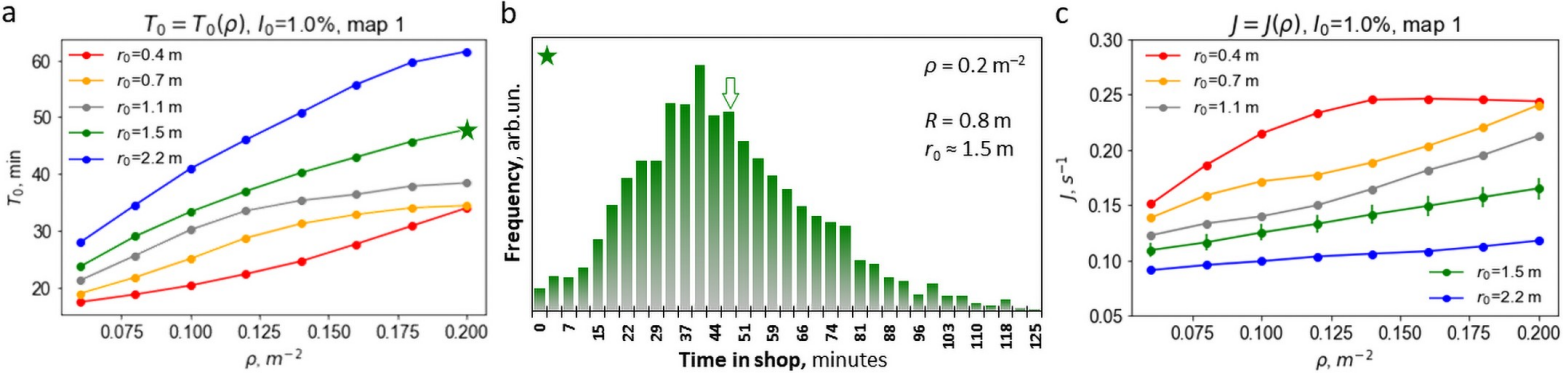
The average time *T*_0_, spent by a customer in the supermarket. (a) *T*_0_ as a function of density *ρ* for different social distances *r*_0_. The star indicates the parameters corresponding to the distribution of average times in panel (b). (b) Distribution of a time spent in the supermarket for *ρ* = 0.2 m^−2^, *r*_0_ ≃ 1.5 m; the arrow indicates the mean time *T*_0_. (c) The average customer flux *J* as a function of the customer density *ρ* for different social distances *r*_0_. Error bars for *r*_0_ = 1.5 m illustrate the typical accuracy for *J*.

For fixed parameters, such as the geometry and area of the shopping room, number of cashiers and time spent at the cash desk, the average time *T*_0_ is some function of the density *ρ* and desired social distance *r*_0_. Similarly, the distribution of *T*_0_ (as the random quantity) is also a function of these quantities. The graphs in the Fig 4a illustrate the according dependence *T*_0_(*ρ*) at different *r*_0_.

As it may be seen from Fig 4a the average time *T*_0_ spent in the store increases with increasing density and social distance. For instance, for the desired social distance *r*_0_ = 1.5 *m* a three-fold increase of density *ρ* from 0.06 to 0.20 *m*^−2^ results in the increasing time from 24 to 47 minutes. This outcome reflects the fact that the desire of the customers to keep far apart from each other slows down the purchasing process.

In Fig 4b the distribution of the time spent by the purchasers in the supermarket are presented for *ρ* = 0.2 m^−2^, *r*_0_ ≃ 1.5 m. The distributions have non-symmetric form with a long tail for large *T*_0_. The results also indicate that the simulation time of 4 hours is long enough to obtain the representative quantities.

The analysis of the mean time *T*_0_ for different supermarket geometries shows similar dependencies, which indicates that the geometry is not a key property for the studied quantities (for more detail see S5 Fig in S1 Appendix).

### The customer flow

The customer flow defines the economic efficiency of a supermarket. Therefore it is important to understand the factors which impact the current to avoid the unnecessary restrictions which can reduce it. The total number of customers is constant in our simulations. Hence, whenever a purchaser leaves the store, an additional customer appears at the entrance zone, that is, the flux out and the flux in, are equal. In our model we compute the time-average customer flux as the total number of visitors leaving the store (from all checkouts) divided by the entire simulation time *J* = *N*_out_/*T*, as well as the instant current *J*_ins_(*t*) = Δ*N*_out_(*t*, Δ*t*)/Δ*t*. Here Δ*N*_out_(*t*, Δ*t*)/Δ*t* is the number of visitors, leaving the store during the time interval [*t*, *t* + Δ*t*] (Δ*t* was taken 10 min). We are mainly interested in the average flux *J*, depicted in Fig 4c; data for the instant flux *J*_ins_(*t*) is given in S6 Fig in S1 Appendix. Fig 4c illustrates the dependence of *J* on the customer density for different social distances. As it follows from the figure, the average customer current monotonically increases with the customer density and decreases with the social radius. Such qualitative dependence has been expected. Still, one comes to an interesting conclusion. When the density, and hence the number of customers in the supermarket doubles, the customer current increases only slightly, in the range of 10–20% (see also S6a Fig in S1 Appendix). From the fundamental point of view such a weak dependence of the flux on density is in a sharp contrast with the analogous process of common matter—the effusion of a gas into vacuum. Here the flux is proportional to the density (see e.g. [47]). From the practical point of view, these findings imply that the presence of a large number of customers in a shopping area is not optimal, neither for sales efficiency nor for the safety at the pandemic conditions.

### The infection spread in a supermarket

Now we analyze the impact of the key parameters, such as density and the desired social distance on the infection spread in the shopping area. We assume that *I*_0_ is a fraction of initially infected visitors, who may transmit the infection to a healthy person. Hence the number of initially infected customers is *NI*_0_ and of initially healthy—*N*(1 − *I*_0_). In a course of time some healthy visitors become newly infected due to contacts with the initially infected ones; they however do not spread the infection further. In the simulations we keep the fraction *I*_0_ constant—when an initially infected customer leaves the supermarket, a similar customer appears at the entrance. When an initially healthy customer leaves the place, a healthy visitor enters, independently, whether the predecessor got infected or not. We observe that after a transient time of about 80 minutes the system attains a steady state (for more detail see S7 Fig in S1 Appendix). In this state the number of infected customers—the initially infected and newly infected, remains constant. Referring for detail to the supplementary sections IV and V in S1 Appendix we present here the probability to get infected Ξ, defined as a ratio of newly infected and initially healthy visitors:

$$\begin{array}{c} \Xi =\frac{N_{\text{infected}}^{\text{new}}}{N\;(1-I_{0})}. \end{array}$$

**Fig 5:**
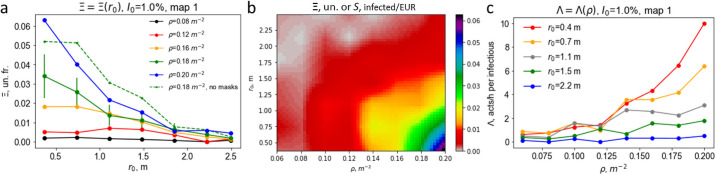
Kinetics of the infection spread in a supermarket. (a) Probability to get infected Ξ as a function of social distance *r*_0_ for different customer densities. Full lines—half of the customers wear medical masks; dashed line—the masks are lacking. Error bars, shown for *ρ* = 0.18 m^−2^, indicate a typical simulation accuracy. (b) Two-dimensional diagram for the probability to get infected Ξ versus *r*_0_ and *ρ*. It coincides with the diagram for the social price *S*(*r*_0_, *ρ*) for the unit profit coefficient, *a* = 1 EUR. For other values of *a* a linear re-scaling is applied (for more explanation see the text). (c) The infection spread rate Λ as a function of *ρ* for different *r*_0_.

The value of Ξ quantifies the risk to go to a supermarket; that is, it gives the probability that a healthy person returns home infected. It is important to know how this risk depends on parameters as *r*_0_ and *ρ*. The dependence of Ξ on the customer density and social distance is given in Fig 5a and 5b.

To assess the effect of wearing medical masks in comparison with the masks absence we performed additional simulations for completely lacking masks for the case of *ρ* = 0.18 m^2^. The results are presented by the green dashed line in Fig 5a. As it may be seen from the figure, the absence of the medical masks leads to about two-fold increase of the risk to get infected.

Another important quantity, which characterizes the infection spread, is the number of customers that get infected from a single initially infected visitor—the infection spread rate Λ:

$$\begin{array}{c} \Lambda =\frac{N_{\text{infected}}^{\text{new}}}{NI_{0}}. \end{array}$$

The dependence of this quantity on *r*_0_ and *ρ* is shown in Fig 5c.

All the results depicted in Fig 5 clearly indicate that the threshold value of the social distance is $r_{0}^{*}≃1.5\;m$. For $r_{0}\leq r_{0}^{*}$ both the probability get infected and the infection spread rate sharply increase with increasing density. At the same time for larger social distances, $r_{0}>r_{0}^{*}$, a very weak dependence of Ξ and Λ on density is observed. The practical consequence of this observation is the following: As long as the condition for the social distance *r*_0_ ≥ 1.5 *m* is fulfilled the density does not have a noticeable impact on the infection spread. Note that the particular $r_{0}^{*}$ value is a product of the choice of model parameters, primarily such as the characteristic length *κ* and the factor *A*^inf^. Although it should not be taken as a result for immediate application in real practice, we expect that the “true” value will not differ much from this one.

### An impact of the supermarket geometry

**Fig 6:**
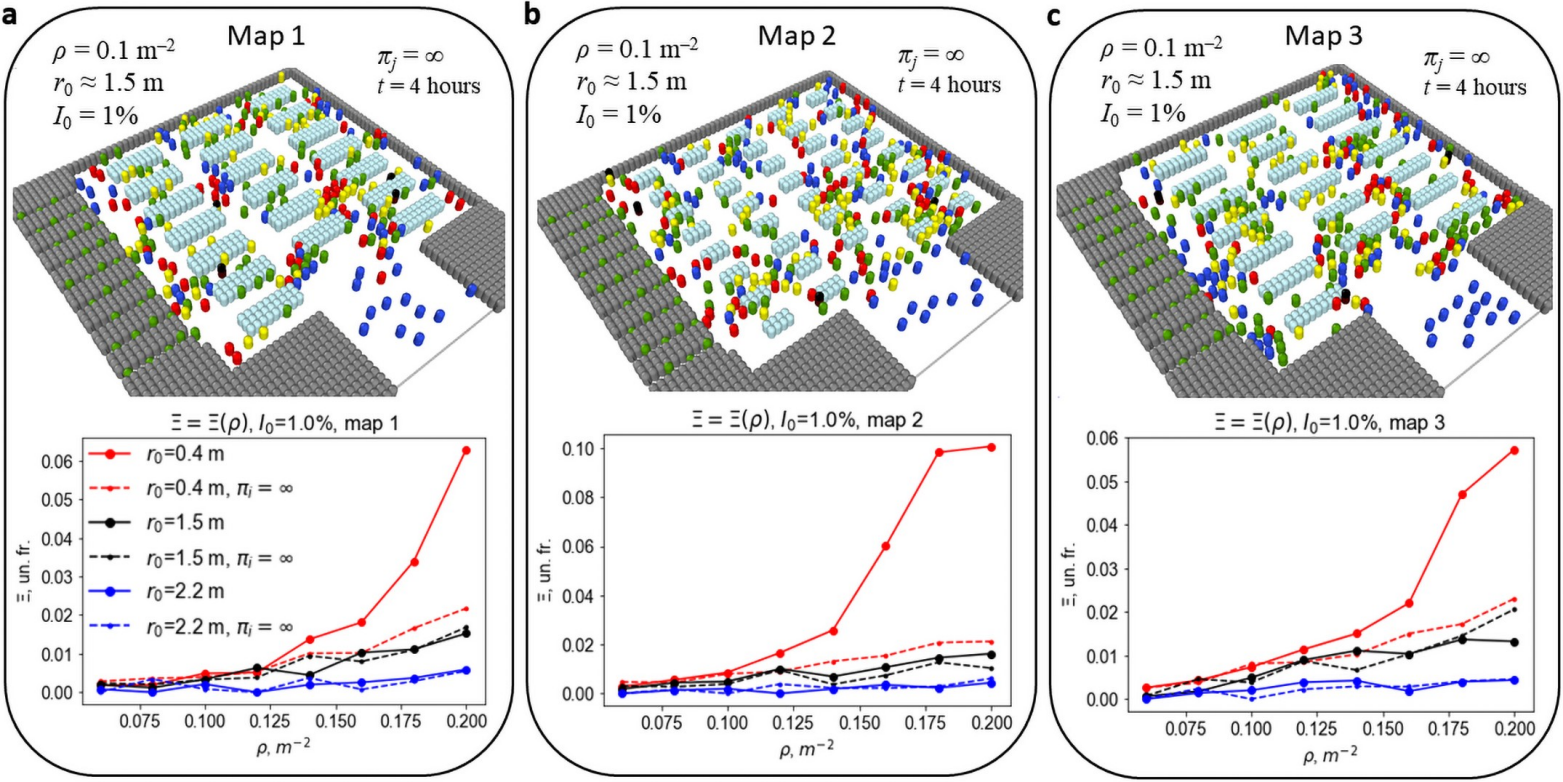
Probability of getting the infection, visiting a model supermarket as a function of *ρ* and *r*_0_ as parameter. The results are presented for different supermarket models: map 1—base (**a**), map 2 (**b**) and map 3 (**c**). Schemes of all the supermarket models are presented in S2–S4 Figs in S1 Appendix. Dashed lines represent results, obtained for simulations without a changing of purchasers behavior in crossroads to Rules B (as if purchases list is infinite *π*_*i*_ = ∞). Significant increase of Ξ with growing *ρ* is observed in all three cases for smallest social distance *r*_0_ = 0.4 m and turned on Rules B. This result can be explained by comparatively large local density of customers near the cashier desks.

To assess an impact of the supermarket geometry on the infection spread we explore two additional supermarket models with different spatial organization. Namely, we vary such important spatial elements as the number of crossroads and width of the thinner passages (“bottleneck” width). The detailed maps are presented in the S1 Appendix. The probability to get infected Ξ as a function of density for different *r*_0_ is shown in Fig 6 for the three studied geometries. As it follows from the figure, for $r_{0}>r_{0}^{*}=1.5\;m$ the infection spread does not depend on the geometry of the shopping place. However for smaller social distances the dependence of Ξ on density becomes significant for the densities larger than 1.3 *m*^−2^. This is another very important conclusion which may be useful for the elaboration of safety rules for crowded places—the geometry of a place is of a minor importance, provided the optimal social distance is kept.

We have also checked an impact of the customer strategy. Namely, we consider the case when the purchase state C (a purchase list is completed) is ignored and a customer always follows the rules associated with the purchase state Ic (a purchase list is not completed). The latter implies that the preferred direction towards the exit, associated with the state C is excluded. While the strategy is not important for large social distances $r_{0}>r_{0}^{*}=1.5\;m$, it becomes important for the smaller ones. This may be explained by the increasing density in the cashier zone due to the customers in the state C. For small social distances this causes the increase of the infection spread. Similar conclusion may be done for the dependence of the social price in the geometry. It is of minor importance as long as the conditions for the optimal social distance are fulfilled, see S1 Appendix.

### The social price

The social price for the supermarket business may be defined as a number of infected people per a unit earned profit. For a customer flux *J*, the average amount of money spent by all customers per unit time reads, *J*〈*π*, where *p* is the average price of one purchase and 〈*π*〉 is the average purchase list per a customer. Then the profit earned per unit time by the supermarket is *αJ*〈*π*, where the coefficient *α* relates the earned money to the profit. At the same time, the number of newly infected customers per unit time is given by Ξ(1 − *I*_0_)*J*, which reflects that only initially healthy customers may be infected. Hence the social price may written as

$$\begin{array}{c} S=\frac{\Xi (1-I_{0})J}{\alpha \;p\;J\;\langle \pi \rangle }=a\;\left(1-I_{0}\right)\;\Xi \approx a\;\Xi , \end{array}$$ (10)

where *I*_0_ has an order of a few percent and we introduce a profit coefficient *a*^−1^ = *αp*〈*π*〉. The dependence of the social price on the social distance and customer density, due to the small value of *I*_0_ in comparison with 1, is almost equal to Ξ(*r*_0_, *ρ*) up to a constant profit coefficient *a*. The social price as a function of the social distance and customer density *S*(*r*_0_, *ρ*) coincides with Ξ(*r*_0_, *ρ*) for the unit profit coefficient, *a* = 1 EUR, see Fig 5b. This figure suggests the policy, how the supermarket business should be organized in order to pay the minimal social price. For instance, if the social price of *S* = 0.01 inf/EUR seems to be acceptable, the customer density *ρ* should not exceed 0.12 m^−2^, together with social distance *r*_0_, not less than 1.5 m. When the average density increases up to 0.2 m^−2^, the social distance should be not less than ∼1.8 m, etc. Certainly, high densities are not recommended, as with increasing densities it is very difficult for the customers to maintain the desired social distance.

## Conclusions

An infection spread in a crowded place—supermarket was investigated, using a composite model comprising three key components: (i) model of inter-customer interactions within a paradigm of social forces, (ii) model of customer purchasing strategy, and (iii) model of the infection transmission. An extensive numerical simulations were performed with the use of standard social force model, which describes the intention of a person keep apart from another one. It mimics his/her perception of the inter-personal distance and the according actions to keep the distance above a desired threshold. This distance is determined, in its turn, by the parameter of the social force *R*—the social radius. The larger *R* the larger the threshold distance. In our simulations we explore a wide range of *R*—from the standard value, to much larger values, which mimics the conscious intention of people to be further from one another for the safety reason.

We introduce a model-independent, objective criterion—the social distance *r*_0_, which may be experimentally measured. It is defined by the location of the first peak of the distribution function *g*_2_(*r*) of the inter-customer distances. We observe that for the range of parameters used in our study *r*_0_ is mainly determined by the social radius *R* and hence is under a conscious control of the customers.

Three main qualitative conclusions follow from our study. Firstly, the infection spread rate is determined by the desired social distance *r*_0_ (which is under a customer control) and only weakly depends on the spatial density of customers. This holds true for a wide range of densities, provided the social distance is larger than the optimal one, which is $r_{0}^{*}≃1.5\;m$ for our model. Secondly, the infection spread rate is practically independent of the geometry of the public space and of the purchase strategy, provided the social distance exceeds the optimal one. Thirdly, the customer flux through the supermarket—the quantity characterising sales efficiency depends rather weakly on the number of customers in the supermarket. This implies the possibility to increase the safety of the customers, by decreasing their number in the supermarket, without sacrificing the business. We introduce the parameter which quantifies the social price for supermarket business; it is equal to the ratio of the number of infected per unit time supermarket visitors to the earned per unit time profit due to all supermarket visitors. The dependence of this quantity on the social distance and customer density is also explored.

Although a complete statistical data for the model calibration is presently lacking, we believe that the chosen parameters are quite realistic. We also believe that the qualitative results reported here are important and will be the base for the elaboration of the scientifically-justified safety rules for other public places, such as transfer/transport hubs, airports, hospitals, offices, etc.

## Supporting information

S1 Appendix Supporting information file—Contains all the supporting schemes and diagrams (S1–S7 Figs). (PDF)
