# Supplementary material for: How risky is it to visit a supermarket during the pandemic?
Source: PLoS One. 2021 Jul 1;16(7):e0253835. doi: 10.1371/journal.pone.0253835 (PMC8248742; doi:10.1371/journal.pone.0253835)
Supplement: S1 Appendix — (PDF) [file pone.0253835.s001.pdf]

How risky is it to visit a supermarket during the pandemic?

Alexey A. Tsukanov[1], Alexandra M. Senjkevich[1], Maxim V. Fedorov[1] and Nikolai V. Brilliantov[1,2]*
[1] *Center for Computational and Data-Intensive Science and Engineering, Skolkovo Institute of Science and Technology, 121205, Moscow, Russia.*
[2] *Department of Mathematics, University of Leicester, Leicester LE1 7RH, United Kingdom.*
*Corresponding author e-mail: n.brilliantov@skoltech.ru, nb144@leicester.ac.uk

Customer strategy

The change of the desired velocity of a customer takes place in the crossroad zones. When a customer enters a crossroad zone and makes a decision to turn of the main text), he/she does it through a double turn, changing the direction two times: Firstly, he/she changes a direction of $\mathbf{v}_{des}$ by the angle 45° at the entrance of the crossroad zone, secondly, he/she turns again by the angle 45° at the exit from the zone. This results in a complete turn by 90°. In the crossroad zone $\mathbf{v}_{des}$ has a diagonal direction with respect to the cardinal directions, see the S1(a) Fig.

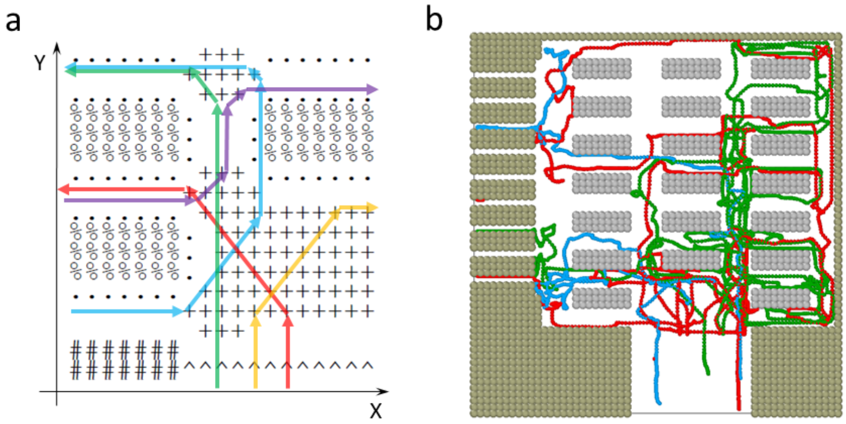

S1 Fig: Customer trajectories in the shopping area. (a) Schematic illustration of the customers behavior in the crossroad zones. The examples of the desired (not actual) trajectories are shown. The following notations are used: # – walls, % – shelving with goods, + – crossroad zone, . – slow motion zone, ∧ – only-forward motion zone. (b) Three typical actual trajectories of visitors for 1 hour of simulation for the Map 1 (base), $\rho = 0.1$ m^{-2}, $r_0 = 1.5$ m. Note that the actual trajectories differ from the desired ones, due to interactions with other visitors.

Supermarket models

Here we present in detail three supermarket models, including maps, which characterize the geometry and the functional parts of the place. The supermarkets differ by the space organization, by the number of crosses and the width of narrowest passages $h_{\rm m}$ ("bottlenecks") as it is depicted in schemes S2-S4 Figs. For the representational convenience we use the reference length, $\Delta_{\rm map} = 1.2$ m and measure the size of the area $L_x \times L_y$ in $\Delta_{\rm map}$ units.

The parameters of the supermarkets are the following:

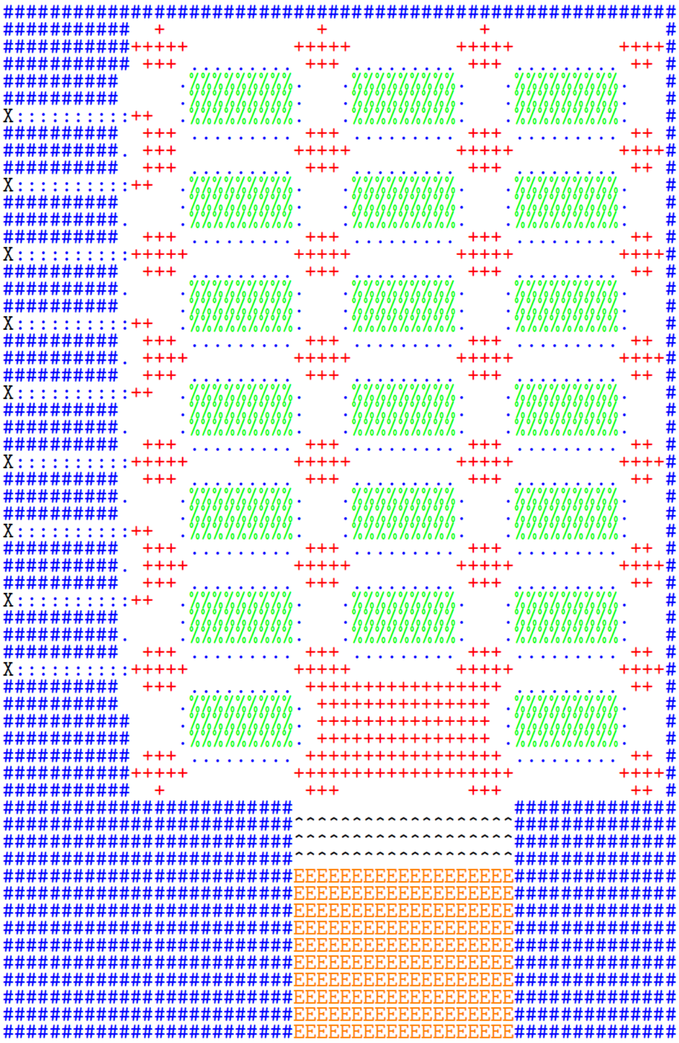

S2 Fig: The scheme of the base supermarket model (Map 1), used in the simulations. Each symbol represents a square zone of $\Delta_{\text{map}} \times \Delta_{\text{map}} = 1.2 \times 1.2\ m^2$. The according notations are: # – walls, % – shelving with goods, E – entrance, + – crossroad zone, . – slow motion zone, : – cashier zone, queue, X – exit, $\wedge$ – only forward motion zone.

Map 1 (base)

$L_x = 58\ \Delta_{\text{map}}$
$L_y = 60\ \Delta_{\text{map}}$
Shopping area $S_{\text{shop}} = 2741.8$ m^2

Free motion zones $S_f = 986.4$ m^2
Number of crosses $n_c = 32$
Bottleneck width (minimum passage width) $h_{\text{m}} = 3.6$ m
Walls area ("#"): 1478.9 m^2
Shelving with goods ("%"): 777.6 m^2
Crossroads area ("+") $S_c = 578.9$ m^2
Slow motion zones (".") $S_s = 691.2$ m^2
Cashier zone, queue (":") $S_q = 129.6$ m^2
Entrance zone ("E", "$\wedge$") $S_e = 355.7$ m^2
Queue (max) length = 12 m
Number of cashier desks ("X") $n_c = 9$

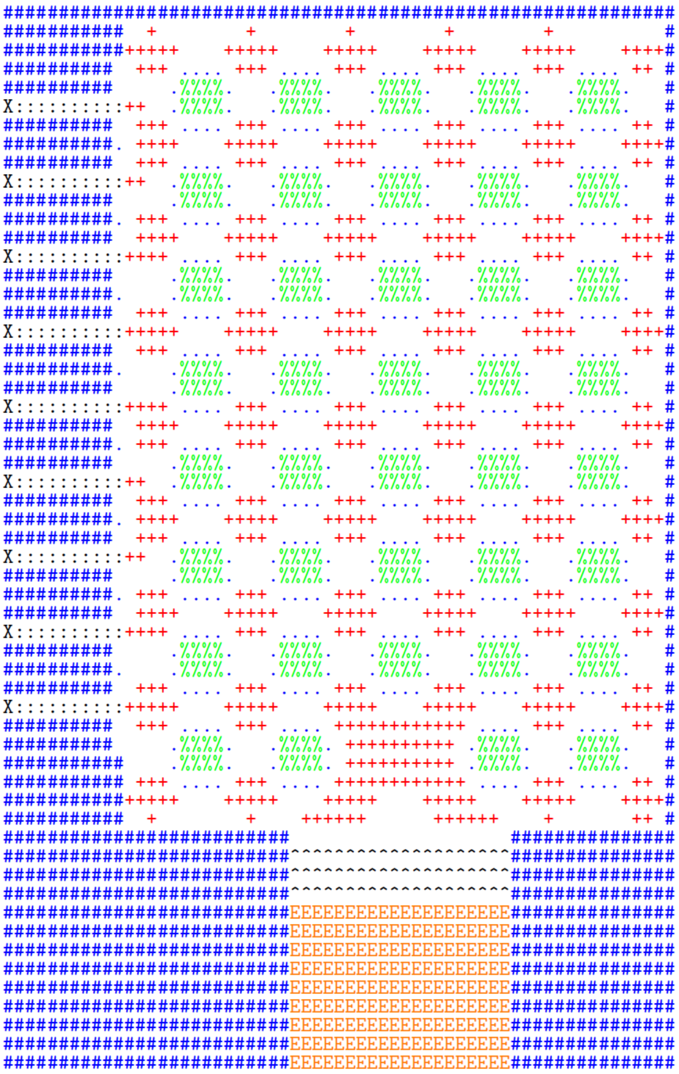

S3 Fig: The scheme of the supermarket model 2, used in the simulations. Meaning of each symbol is similar to scheme in S2 Fig.

The geometry of the supermarket (base model) is shown in S2 Fig ("Map 1"). The according zones are encoded in the maps by the respective symbols.
Note, that the total shopping area is a sum $S_{\mathrm{shop}} = S_f + S_c + S_s + S_q + S_e$. That is, the walls and obstacles (shelving with products) are not included.

Map 2

$L_x = 61\ \Delta_{\mathrm{map}}$
$L_y = 57\ \Delta_{\mathrm{map}}$
Shopping area $S_{\mathrm{shop}} = 3114.7\ \mathrm{m}^2$
Free motion zones $S_f = 1111.7\ \mathrm{m}^2$
Number of crosses $n_c = 54$
Bottleneck width $h_{\mathrm{m}} = 3.6$ m
Walls area: $1429.9\ \mathrm{m}^2$
Shelving with goods: $449.3\ \mathrm{m}^2$
Crossroads area $S_c = 853.9\ \mathrm{m}^2$

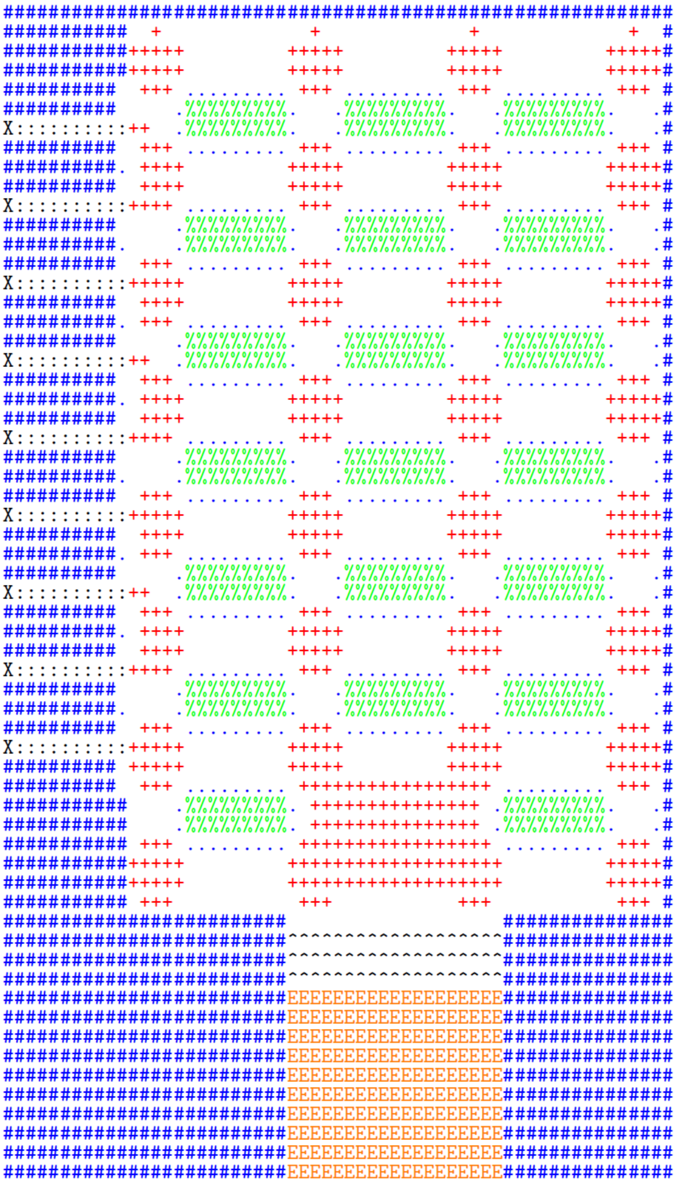

S4 Fig: The scheme of the supermarket model 3. Meaning of each symbol is similar to scheme S2 Fig.

Slow motion zones $S_s = 673.9\ \mathrm{m}^2$
Cashier zone, queue $S_q = 129.6\ \mathrm{m}^2$
Entrance zone $S_e = 345.6\ \mathrm{m}^2$

The geometry of the supermarket model ("Map 2") is shown in scheme S3 Fig.

Map 3

$L_x = 59\ \Delta_{\mathrm{map}}$
$L_y = 61\ \Delta_{\mathrm{map}}$
Shopping area $S_{\mathrm{shop}} = 3133.4\ \mathrm{m}^2$
Free motion zones $S_f = 1167.8\ \mathrm{m}^2$
Number of crosses $n_c = 32$

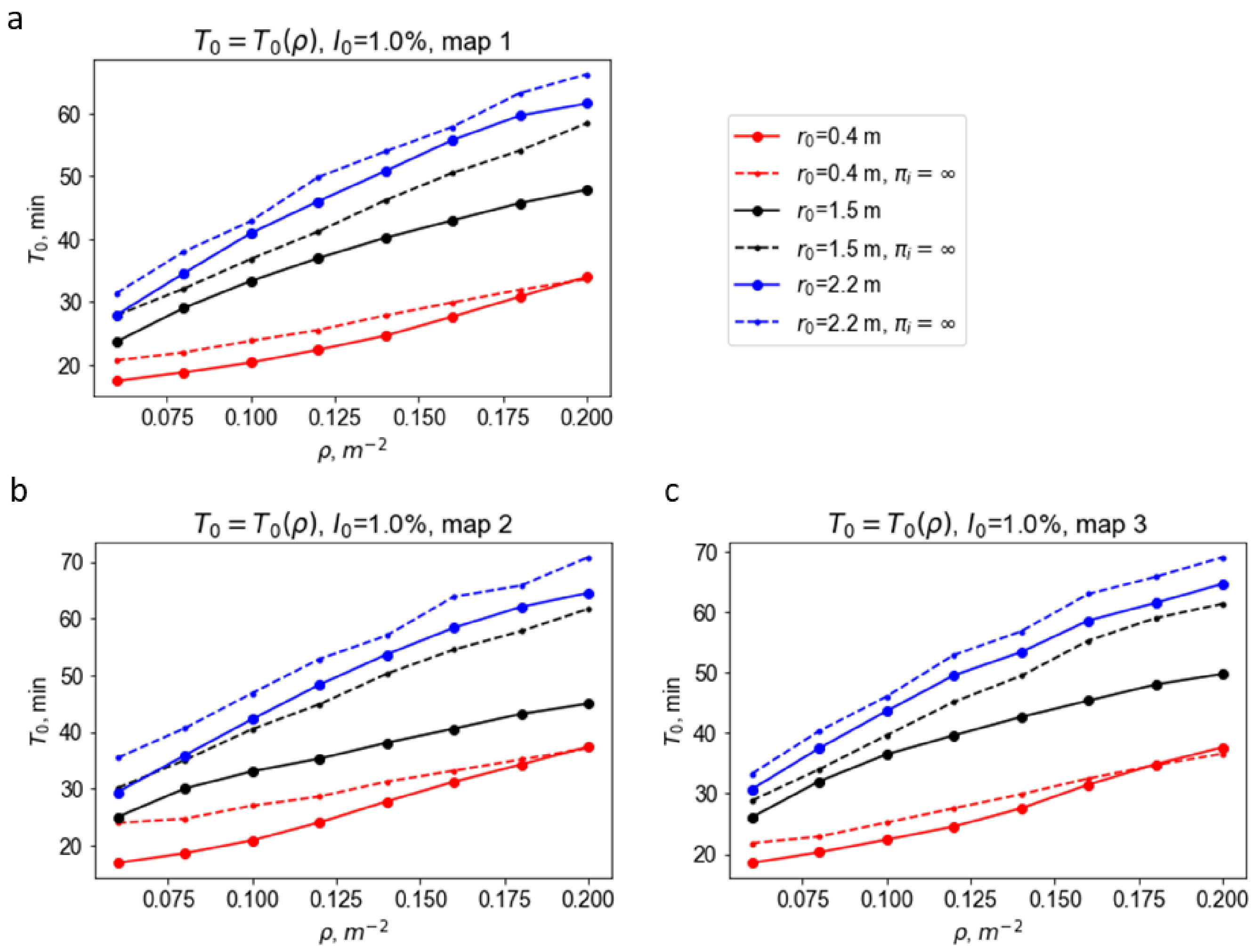

S5 Fig: The mean time T_0, spent in the supermarket, as a function of the customer density ρ at three different values of social distance r_0. The results refer to three different supermarket models: (a) Map 1 (base), (b) Map 2 and (c) Map 3. Dashed lines correspond to the results, obtained for the simulations for the case of $\pi_i = \infty$, that is, for the case of a permanent purchase behavior.

Bottleneck width h_m = 4.8 m
Walls area: 1517.8 m^2
Shelving with goods: 518.4 m^2
Crossroads area S_c = 826.6 m^2
Slow motion zones S_s = 653.8 m^2
Cashier zone, queue S_q = 129.6 m^2
Entrance zone S_e = 374.4 m^2

The geometry of the supermarket model ("Map 3") is shown in scheme S4 Fig.

Time spent in the supermarket and customer fluxes

Here we present the distribution of time spent in the three different supermarkets for the customers densities ρ and social distances r_0 (S5 Fig).

Figure S6 Fig illustrates the customers flux J in the supermarket (Map 1) averaged over time, as a function of the social distance r_0 and customer density ρ. The respective two-dimensional plot in S6b Fig illustrates the dependence

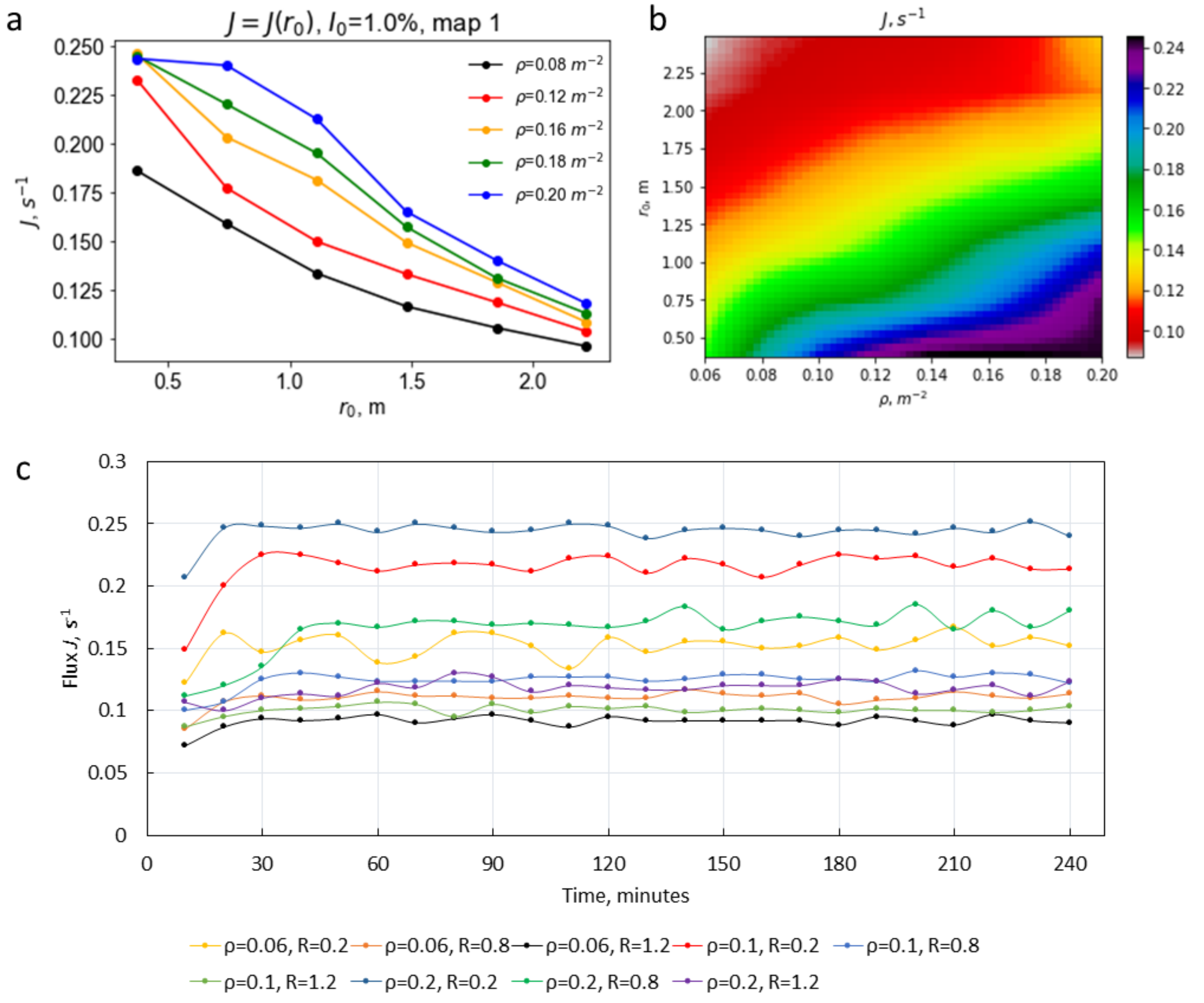

S6 Fig: The average customer flux J. (a) J as a function of the social distances r_0 for different customer density ρ. (b) Two-dimensional plot of J as a function of both r_0 and ρ. Colour bar shows the value of J in units of s^{-1}. (c) Customer flux J as a function of time, steady state regime is reached within 20-40 minutes.

of J on both parameters ρ and r_0.

The dynamics of the cumulative number of infection acts and the number of infected visitors

The dependence of the infection spread and infection rate current on such parameters as the customer density ρ (the number of customers per the shopping area), the social distance r_0 and the percentage of initially infected visitors seems to be of a primary importance. Therefore we analyse the time dynamics of the following indicators: the cumulative number of infection acts and number of infected customers in the whole supermarket area. The results for $\rho = 0.16$ m^{-2}, $r_0 \approx 1.5$ m and different percentage of initially infected visitors – I_0 0.4%, 1% and 2% are shown in S7 Fig. After a certain initial period of time (about $30 - 60$ min), the accumulated number of infection acts becomes an almost linear function of time. The transmission process can be characterized by such a parameter as number of infection acts per unit time. We will use this parameter calculated per one infectious visitor, that is, how many people can be infected per one hour by one infectious visitor – the infection spread rate Λ.

Due to a permanent influx of healthy visitors, a number of infected ones can never reach 100%. Actually, after

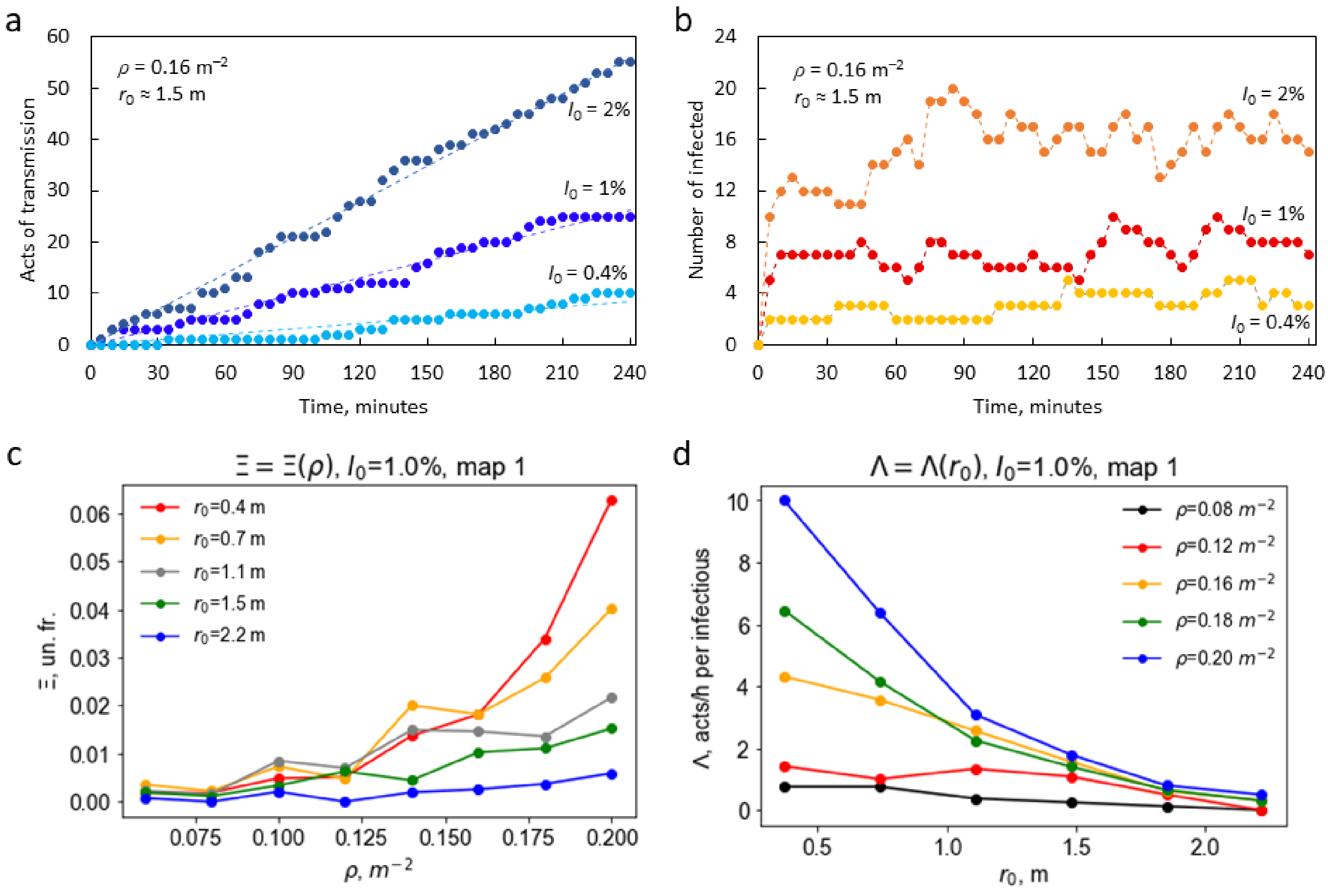

S7 Fig: The dynamics of a cumulative number of infection acts (a) and number of infected people (b) in the supermarket (Map 1) for different percentage of initially infected visitors – $I_0 = 0.4\%, 1\%, 2\%$. The customer density and the social distance are respectively $\rho = 0.16$ m^{-2} and $r_0 \approx 1.5$ m. (c) The probability to get infected Ξ as a function of the customer density ρ at different values of desired social distance r_0, and (d) the infection spread rate Λ, which is the number of customers that get infected from a single initially infected visitor.

about 80 minutes this quantity reaches a steady value and fluctuates around it, see S7b Fig.

Computational efficiency

We used a simple molecular dynamics (MD) scheme (without neighbor lists), with the computational complexity of $\sim N^2$ (squared number of visitors). For numerical simulations, we exploit both, a common laptop as well as the supercomputer. In the latter case, the Message Passing Interface (MPI) was used and parallelization has been deployed. Each CPU core simulated a model with a particular set of major model parameters (multiparameter parallel simulations). The simulation time for a fixed number of time steps depends on the density of visitors ρ. One core simulation for the density $\rho = 0.06\ m^{-2}$ $(N = 165)$ was about 2.5 minutes, for density $\rho = 0.1\ m^{-2}$ $(N = 276)$ – about 7 minutes, for $\rho = 0.2\, m^{-2}$ $(N = 551)$ – less than 30 minutes. The algorithm may be further optimized.

The source code (`C++`) with all necessary data files can be found in GitHub `https://github.com/AATsukanov/Infection-Transmission-Model-2021/`.
